# An Investigation of the Reliability of Different Types of Sensors in the Real-Time Vibration-Based Anomaly Inspection in Drone

**DOI:** 10.3390/s22166015

**Published:** 2022-08-12

**Authors:** Mohamad Hazwan Mohd Ghazali, Wan Rahiman

**Affiliations:** 1 School of Electrical and Electronic Engineering, Universiti Sains Malaysia Engineering Campus, Nibong Tebal 14300, Penang, Malaysia; 2 Cluster of Smart Port and Logistic Technology (COSPALT), Universiti Sains Malaysia Engineering Campus, Nibong Tebal 14300, Penang, Malaysia

**Keywords:** sensors, vibration, drone, noise, anomaly inspection, mobile application

## Abstract

Early drone anomaly inspection is vital to ensure the drone’s safety and effectiveness. This process is often overlooked, especially by amateur drone pilots; however, some faulty conditions are difficult to notice by the naked eye or discover, even though the drone inspection process has been conducted; therefore, a real-time early drone inspection approach based on vibration data is proposed in this study. Firstly, the reliability of several microelectromechanical systems (MEMS) sensors, namely the ADXL335 accelerometer, ADXL 345 accelerometer, ADXL377 accelerometer, and SW420 vibration sensor in detecting faulty conditions, were tested and compared. The experimental results demonstrated that the vibration parameter measured using ADXL335 and ADXL345 accelerometers are the best choice as most of the faulty conditions can be detected, in contrast to other MEMS sensors. The output produced from the anomaly inspection algorithm is then converted to the “Healthy” or “Faulty” state, which is displayed in a mobile application for easy monitoring.

## 1. Introduction

Drones or unmanned aerial vehicles (UAVs) have continuously evolved with exceptional growth over the past fifteen years. Initially, drones were only available for military purposes, but over the last decade, the use of drones for commercial purposes has received lots of attention. Lower prices and higher flexibility are what make drones so popular nowadays as they can be applied to various applications, such as agriculture [1,2], mapping [3,4], traffic monitoring [5,6], and rescue missions [7,8]; however, there is no doubt that drones are prone to crashing, which might lead to unwanted casualties. According to Gorucu and Ampatzidis [9], there were approximately 4250 drone-related injuries from 2015 to 2020 in the USA alone, in which most of the cases are caused by drone crashes.

There are many factors that make drones vulnerable to crashes, such as low battery level, loss of communication with the drone, weather conditions, and faulty drone components. Among the factors mentioned above, a drone crash originating from faulty drone components can be avoided with early drone maintenance. Most drone pilots often overlook the drone maintenance process as it is considered unimportant. In fact, this process can prevent future drone crashes by notifying us regarding the faulty components. For instance, an unbalance propeller or a crack in one of the drone arms can be detected by the early maintenance process based on vibration data.

Among many different solutions concerning anomaly inspection in drones, most of them fall into current, acoustic, and vibration-based approaches. As for the works related to current data, Lee et al. [10] proposed a fault diagnosis technique for UAV motors based on their steady-state condition. An infrared sensor was installed facing the motor to calculate its angular speed, and the current is measured using a current sensor at the entrance of electronic speed controller (ESC). A fault detection and diagnosis model was developed by Saied et al. [11] based on the speeds and electric currents of the brushless motors measured by their ESCs. To simulate a motor and propeller failure, commands are sent to stop the targeted motor at desired times, and the propeller is detached from its corresponding motor spindle before the flight, respectively. In [12], a speed and failure detection approach for brushless motors was developed based on the angular speed and current data. An unbalanced propeller and loose stator motor are simulated to obtain faulty data. Although it was reported that the current-based approach can successfully detect anomalies in drones, it is more suitable to be applied for faulty electrical components such as the motor and ESC.

A sound-based condition monitoring method can detect more faults than a current-based one, and acquiring sound signals is easier, according to Altinors et al. [13]. Iannace et al. [14] applied the noise emitted by the drone to develop a neural network (NN) model to detect faulty propeller blades. Another sound-based approach was performed by Rangel-Magdaleno et al. [15], who implemented the discrete wavelet transform (DWT) and Fourier transform techniques to detect balance in the drone propellers. Pechan and Sescu [16] performed experimental work to analyze the noise radiating from several propellers that have different surface imperfections. The sound-based technique has a major disadvantage, in which it is susceptible to background noise. The sound samples can also be affected by sounds from other motors.

According to Rafiee et al. [17], vibration extensively exists in rotating machinery, and vibration-based techniques are widely used in the condition monitoring of rotating machinery. Bondyra et al. [18] presented an anomaly detection technique for UAV rotor blades. Based on the acceleration data in x, y, and z-directions obtained from the onboard Inertial Measurement Unit (IMU ADIS16488), the support vector machine (SVM) technique is used to classify the signals and determine the presence and scale of a fault on the rotor blades. In [19], no external vibration sensors are used to detect faults in multirotor propellers. Instead, the built-in accelerometer is utilized to measure the vibration signals, where the location of a damaged propeller can be identified in the offline mode, but limited by the flight trajectories. Pourpanah et al. [20] used a combination of external accelerometers and current sensors to classify between healthy and broken propellers (normal, 5%, 10%, or 15% broken), based on vibration and current signals. A healthy propeller generates a smooth signal compared to a broken propeller.

Considering a large volume of data generated from different operating conditions under different drone configurations, a process that combines data from multiple sources is vital. Work regarding data fusion has been performed by Zhang et al. [21] in the safety distance diagnosis of drones, which is essential in the line inspection application. An adaptive weighted data fusion technique was adopted to combine several parameters that contribute to the determination of the safe distance of the UAV line. Gultekin et al. [22] proposed a convolutional neural network (CNN)-based data fusion technique for fault detection of autonomous transfer vehicles (ATVs), which utilizes the time–frequency information of vibration signals. The decision-making process regarding the ATV’s condition is engaged after fusing the outputs of CNN. Meanwhile, Guo et al. [23] fused the data from accelerometers, gyros, wind vanes, and global positioning system to detect faults in the airspeed measurement systems of UAVs. Previous studies demonstrated the capability of data fusion in providing more valuable and reliable information compared to single sensor approaches.

The real-time anomaly detection techniques often utilize one or more microelectromechanical systems (MEMS) sensors in their framework. MEMS sensors are microstructures capable of coupling the mechanical and electrical aspects at the microscale level, which results in an amplified coupling efficiency [24]. the MEMS sensor is suitable to be applied in applications related to drones due to its lightweight feature, where it can be equipped to the drone during flying. MEMS sensors also have been widely applied in low-cost vibration monitoring tasks. Sukenda et al. [25] integrated the SW420 vibration sensor with the Arduino UNO to detect the presence of a train. The vibrations on the rails induced by the incoming train can be sensed by the MEMS sensors and the vibration output is displayed on the liquid-crystal display (LCD). A similar sensor is also applied by Bhuiyan et al. [26] and Pakpahan et al. [27] in a machine’s vibration monitoring. It has been demonstrated in both works that the SW420 vibration sensor can successfully detect vibration and further differentiate the levels of vibration.

An ADXL335 accelerometer was integrated with Arduino UNO to measure the vibration level of a test vehicle for road maintenance purposes [28]. In [29], an ADXL335 accelerometer was utilized for anomaly detection in induction motor based on vibration level. Bansal and Vedaraj [30] used an ADXL345 accelerometer and Arduino UNO in machine vibration monitoring during turning operation. A similar MEMS sensor has also been applied by Soother et al. [31] in the vibration monitoring of induction motor. Kulkatni and Cheeran [32] demonstrated a low-cost vibration monitoring of the printed circuit board using ADXL377 accelerometer. Based on the reported literature, apart from the SW420 vibration sensor, MEMS accelerometers also excel in vibration-based anomaly inspection applications.

Most of the studies performed by other researchers are offline methods, where the condition monitoring and decision-making are not in real-time. Offline methods might take a longer time to determine the drone’s condition, and the decision-making process cannot be carried out on the spot. In addition, a computer or laptop is usually necessary for the offline method to analyze the drone data and make a decision. This makes the offline method not feasible for an amateur drone pilot. In contrast to those previous studies that mostly cover the faulty motor and propeller cases, this paper presents a real-time vibration-based early anomaly inspection in a drone that focuses on propeller and drone arm components. The main contribution of this study is the performance evaluation of several MEMS vibration sensors in online anomaly inspection of drone components based on the vibration data. We also created a mobile application, where users can inspect the drone condition based on the healthy and faulty indicators.

This paper is organized as follows: Section 2 presents the different MEMS sensors applied in this study and the vibration output produced for each configuration of drone components. The proposed anomaly inspection system and noise reduction are presented and discussed in Section 3. We presented the mobile application for easy user interface and monitoring in Section 4. Finally, in Section 5, we conclude the paper and discuss future works.

## 2. MEMS Sensors Performance Comparison

As mentioned in Section 1, four different sensors are utilized for the early drone maintenance process, one at a time. The sensors involved are the SW420 vibration sensor, ADXL345 accelerometer, ADXL335 accelerometer, and ADXL377 accelerometer. The advantages and disadvantages of each sensor are listed in Table A1 in Appendix A. Each sensor is connected to the Arduino DUE to collect and classify healthy and faulty drone components. The drone used in this study is the Storm Drone 8, which is an octocopter-type drone.

In this study, the MEMS sensors selected are the widely used ones, based on screening several related studies conducted by other researchers. Referring to the Introduction, the SW420 vibration sensor and ADXL345 accelerometer are the most popular MEMS sensor. Next, different technical specifications are considered in the MEMS sensor selection, such as weight, interface, and g-range. The final selected MEMS sensors are SW420, ADXL335, ADXL345, and ADXL377. Due to limited availability in the market, the MEMS displacement and velocity sensors are not included in our study. All the MEMS sensors are mounted at the drone arm and as close to the brushless motor as possible. For the initial stage, we evaluate the performance of each MEMS sensor in capturing the vibration data and detecting any anomalies. For the accelerometers, only acceleration values in the x-direction are observed because anomalies can still be detected with one axis. The maximum and average amplitude values are utilized to differentiate the healthy and faulty drone components. After comparing the performance of all sensors and determining the best MEMS sensors to be applied, the remaining seven sensors are installed at each drone arm. In total, at the final stage, there are eight MEMS sensors installed at eight drone arms. All vibration data are collected in an indoor environment, and when the drone is in the “warm-up stage”, which is the stage in which propellers spin before taking off.

### 2.1. Drone’s Components Configuration

**Figure 1:**
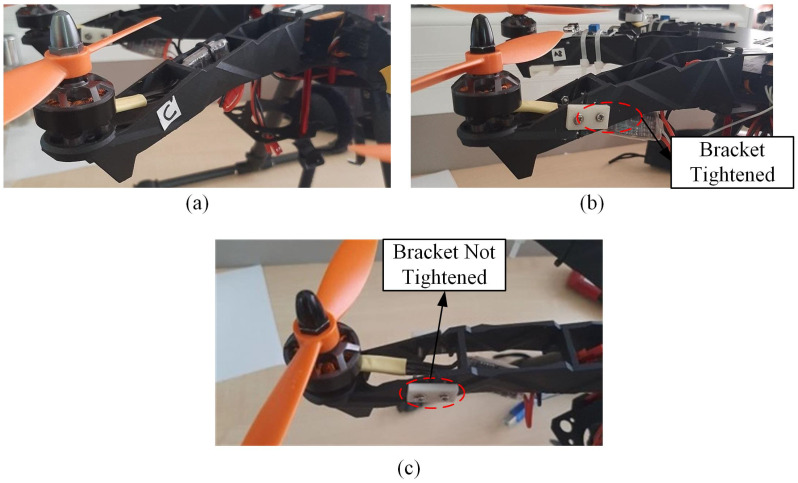
The drone arm configurations involved in the experiment: (**a**) normal arm, (**b**) arm with a minor crack, and (**c**) arm with a major crack.

The drone’s arm and propeller are considered in the experimental study. The vibration data are collected in the healthy and faulty arm and propeller conditions to evaluate the reliability of the selected MEMS vibration sensor. The configurations for the drone arm component can be categorized into a healthy or normal arm, an arm with a minor crack, and an arm with a major crack. A crack in the drone arm occurs due to the previous crash of the drone and is often overlooked by drone pilots. Referring to Figure 1, the drone arm is modified by cutting and joining back using plastic brackets to simulate the minor and major cracks. This bracket can be tightened and loosened to represent minor and major cracks, respectively.

**Figure 2:**
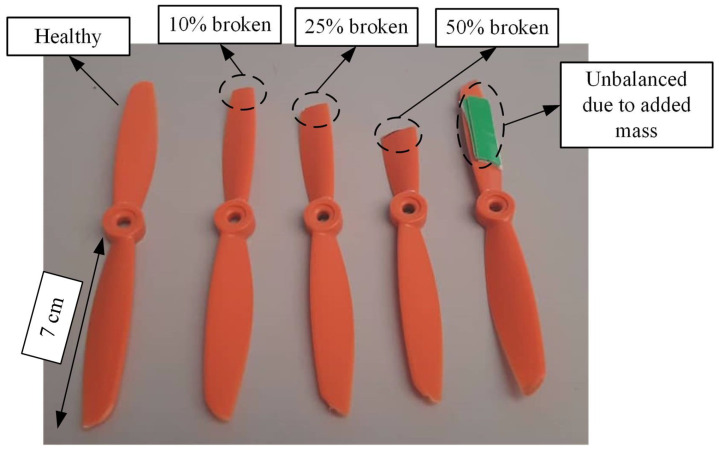
The healthy and faulty propeller samples used in the experiment.

The faulty propeller can be differentiated into 10% broken, 25% broken, 50% broken, and unbalanced propellers due to added weight. These conditions can be seen in Figure 2. The propellers are broken into three different broken rates to determine the strength of vibration induced by each faulty propeller. At first, the SW420 and ADXL377 sensors are unable to detect vibration induced by the 10% and 25% broken propellers, which are discussed in the later subsection of this manuscript. Thus, for the purpose of performance evaluation, we increase the broken rate to the point where SW420 and ADXL377 sensors can detect the vibration. As a result, 50% is the broken rate of the propeller that can be detected by SW420 and ADXL377 sensors. The broken rates are determined based on the length of the propeller blade. In this case, the propeller blade’s length is 7 cm, as shown in Figure 2. Thus, 10%, 25%, and 50% broken rates correspond to the 0.7 cm, 1.75 cm, and 3.5 cm of the propeller being cut.

### 2.2. SW420 Vibration Sensor

**Figure 3:**
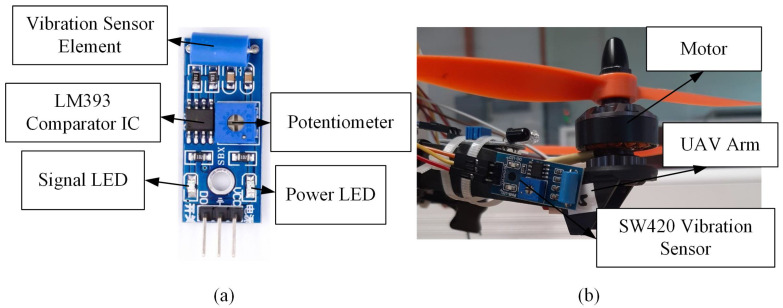
(**a**) The SW420 vibration sensor; (**b**) the mounting position of the SW420 vibration sensor on the drone arm.

The SW420 vibration sensor is a non-directional sensor that produces two types of output: analog output (values are in the form of received voltages) and digital output (based on values 0 and 1). The analog output was utilized in this study, where a higher value corresponds to a higher vibration amplitude. The sensitivity of the SW420 vibration sensor can be adjusted by turning the onboard potentiometer [33], as shown in Figure 3. The power LED will turn on when connected to the power supply, whereas the signal light emitting diode (LED) will flash if there is a vibration. The LM393 comparator integrated circuit (IC) compares the reference voltage and the input vibration signal before relaying the values to the digital output pin in the form of binary states. The technical specification of this sensor can be seen in Table 1.

A Kalman filter was implemented to reduce noise obtained from raw sensor readings by adjusting the currently measured sensor value based on the past sensor data [34]. In this case, the Kalman filter was only used to filter the raw data outputs of the SW420 vibration sensor due to the high presence of noise from this sensor compared to the accelerometers. The Kalman filter can be described by the following equations:

(1) $$x_{k}=Ax_{k-1}+Bu_{k-1}+w_{k}$$

(2) $$z_{k}=Hx_{k}+v_{k}$$

where *x*, *u*, and *z* are state, feedback, and measurement or observation vectors, respectively. The status transition matrix *A* relates the state at time step k−1 to the state at step *k*, whereas the feedback matrix *B* relates the control input *u* to the state *x*. The measurement matrix *H* relates the state $x_{k}$ to the measurement $z_{k}$. $w_{k}$ and $v_{k}$ represent the process and measurement noises, respectively.

The Kalman filter algorithm can be classified into state prediction (time update) and correction (measurement update) algorithms. The state prediction algorithm applied is based on the following equations:

(3) $${\hat{x}}_{k}^{-}=A{\hat{x}}_{k-1}+Bu_{k-1}$$

(4) $$P_{k}^{-}=AP_{k-1}A^{T}+Q$$

where ${\hat{x}}_{k}^{-}$ is the priori state, estimated before the measurement update correction, whereas $P_{k}^{-}$ is the prior covariance matrix. *Q* refers to the process noise covariance matrix. The correction algorithms are defined as follow:

(5) $$K_{k}=P_{k}^{-}H^{T}{\left(HP_{k}^{-}H^{T}+R\right)}^{-1}$$

(6) $${\hat{x}}_{k}={\hat{x}}_{k}^{-}+K_{k}\left(z_{k}-H{\hat{x}}_{k}^{-}\right)$$

(7) $$P_{k}=\left(I-K_{k}H\right)P_{k}^{-}$$

where $K_{k}$, $P_{k}$, *R*, ${\hat{x}}_{k}$, and *I* are the Kalman gain, the posteriori error covariance matrix, the covariance matrix of the measurement noise or filter deviation matrix, the estimate of *x* at time *k* (the optimum filter value), and the unit matrix, respectively. The process of prediction and correction occurs continuously to generate the estimated measurement value; thus, the measurement noise that appears in the sensor system can be reduced. The measurement using the SW420 vibration sensor are obtained at 40 Hz sampling frequency and 0.01 Hz frequency resolution.

**Figure 4:**
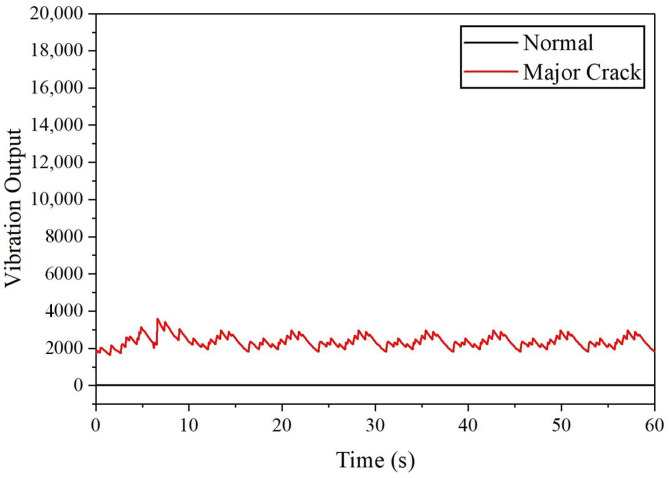
The vibration output acquired from SW420 vibration sensor for drone arm fault simulations (major crack).

**Figure 5:**
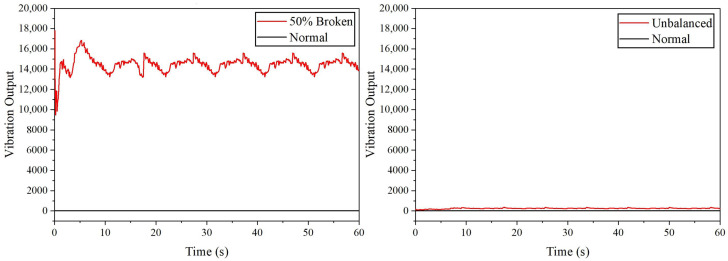
The vibration output acquired from SW420 vibration sensor for propeller fault simulations.

For drone arm configurations, it was found that the minor crack in the drone arm cannot be detected by the SW420 vibration sensor due to the 0 vibration output recorded; however, referring to Figure 4, the major crack in the drone arm can be successfully detected as the maximum amplitude level obtained is 3495. For propeller fault simulation, each faulty configuration is compared with the healthy one to detect any anomaly. The vibration output recorded for healthy, 10% broken, and 25% broken propellers are 0, which means that the vibration induced by these propellers cannot be detected by the SW420 vibration sensor. From Figure 5, it can be observed that the SW420 vibration sensor can only detect anomalies that come from the unbalanced and 50% broken propeller. In terms of maximum vibration level, 50% broken propeller produces the highest vibration amplitude of 16,832, followed by an unbalanced propeller, as listed in Table 2.

### 2.3. ADXL345 Accelerometer

**Figure 6:**
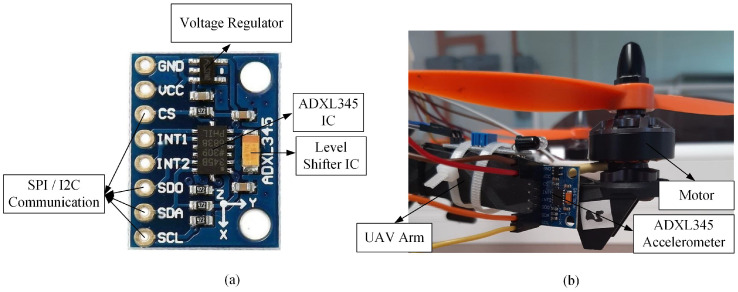
(**a**) The ADXL345 accelerometer; (**b**) the mounting position of the ADXL345 accelerometer on the drone arm.

ADXL345 (as shown in Figure 6) is a low-power, 3-axis MEMS accelerometer that can detect static and dynamic accelerations. This accelerometer consists of fixed and moving plates, which can deflect in any direction when subject to acceleration in the *x*, *y*, and *z*-direction. This deflection causes a change in capacitance on each axis and is then converted to an output voltage proportional to the acceleration. The unit measured using this sensor is g and can be converted into hertz (Hz) unit. The ADXL345 accelerometer is equipped with both inter-integrated circuit (I2C) and serial peripheral interface (SPI) interfaces. The onboard voltage regulator and level shifter IC make this sensor compatible with a 5 V microcontroller. The technical specifications of the ADXL345 accelerometer are listed in Table 3.

**Figure 7:**
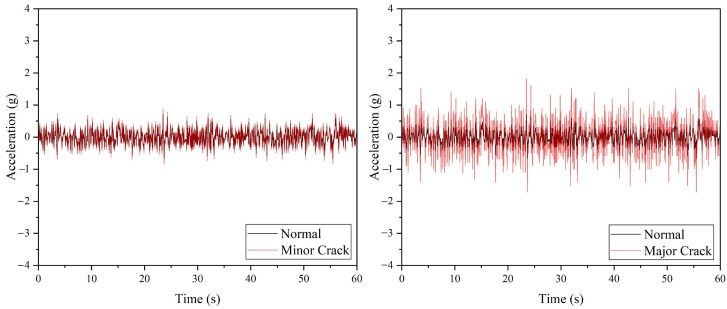
The acceleration values acquired from ADXL345 accelerometer for drone arm fault simulations.

**Figure 8:**
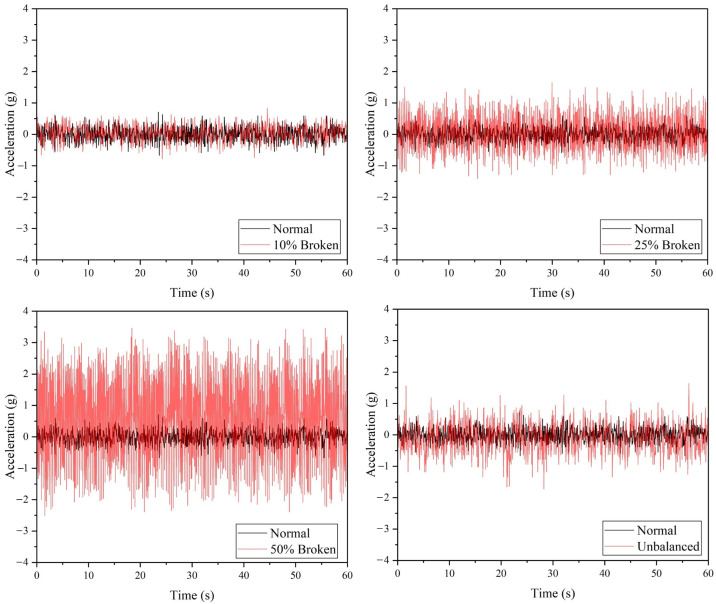
The acceleration values acquired from ADXL345 accelerometer for propeller fault simulations.

The anomaly inspection for drone arm configurations using the ADXL345 accelerometer is depicted in Figure 7, where a minor and major crack in the drone arm can be successfully detected. For the minor crack case, the discrepancy in the maximum amplitude and average absolute amplitude values between the normal arm and arm with minor crack is minimal, as listed in Table 4. For the propeller fault cases, compared to the SW420 vibration sensor, all propeller faults can be detected by the ADXL345 accelerometer, as shown in Figure 8; however, similar to the minor crack case, it is recommended to inspect the propeller several times due to the slight differences in the maximum amplitude and average absolute amplitude values of the 10% broken propeller compared to the normal one.

### 2.4. ADXL335 Accelerometer

**Figure 9:**
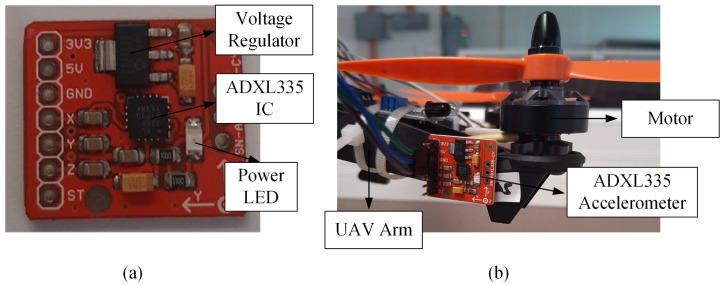
(**a**) The ADXL335 accelerometer; (**b**) the mounting position of the ADXL335 accelerometer on the drone arm.

The ADXL335 (as shown in Figure 9) is a low-power sensor with an integrated signal conditioning circuit that can measure static and dynamic acceleration within the range of ±3 g. The working mechanism is similar to the ADXL345, where a deflection of the moving mass will result in a sensor output whose amplitude is proportional to the acceleration. The technical specifications of the ADXL345 accelerometer are listed in Table 5.

**Figure 10:**
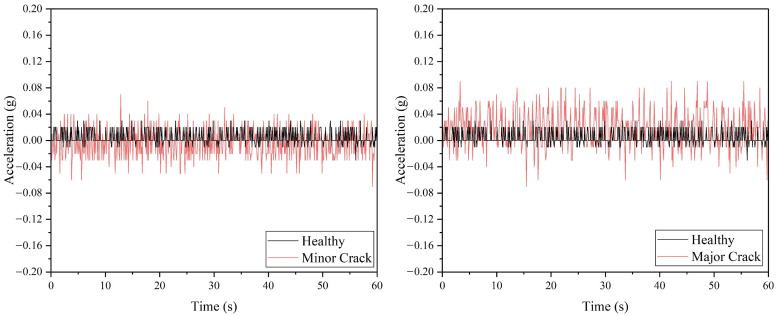
The acceleration values acquired from ADXL335 accelerometer for drone arm fault simulations.

**Figure 11:**
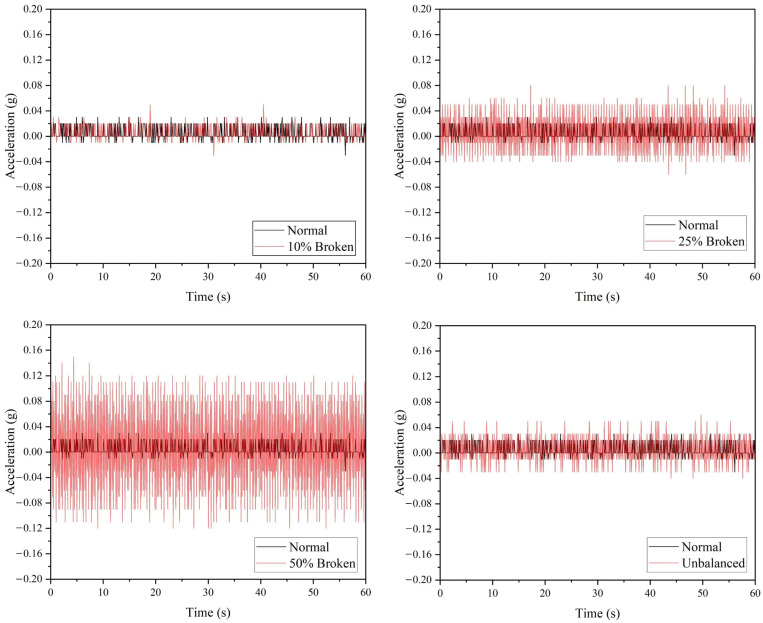
The acceleration values acquired from ADXL335 accelerometer for propeller fault simulations.

Figure 10 demonstrated the performance of the ADXL335 accelerometer, where a drone arm with minor or major crack can be detected based on the raw acceleration values. Referring to Table 6, the maximum vibration amplitude and average absolute amplitude recorded for the minor and major crack arm are greater than the values of the normal drone arm. Similar to the ADXL345 accelerometer, the ADXL335 accelerometer can detect all the faulty propeller cases based on the raw acceleration data, as shown in Figure 11. The vibration amplitude produced by the 50% broken propeller is the highest (0.15 g), followed by 25% broken (0.08 g), unbalanced (0.06 g), and 10% broken propellers (0.03 g).

### 2.5. ADXL377 Accelerometer

**Figure 12:**
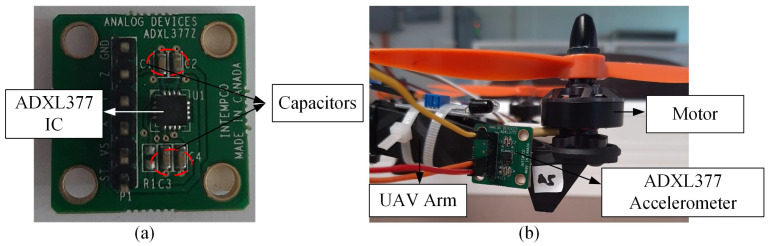
(**a**) The ADXL377 accelerometer; (**b**) the mounting position of the ADXL377 accelerometer on the drone arm.

Similar to the ADXL335 accelerometer, the ADXL377 (as shown in Figure 12) is a 3-axis analog-output accelerometer that can measure more extreme changes in static and dynamic acceleration with a ±200 g measurement range. According to the datasheet, the ADXL377 accelerometer comes with four factory-installed capacitors (C1, C2, C3, and C4). C1 capacitor is a bypass capacitor to reduce supply noise, whereas C2, C3, and C4 are filter capacitors (10 nF) that can be changed to set the analog bandwidth. The technical specifications of the ADXL345 accelerometer are listed in Table 7.

**Figure 13:**
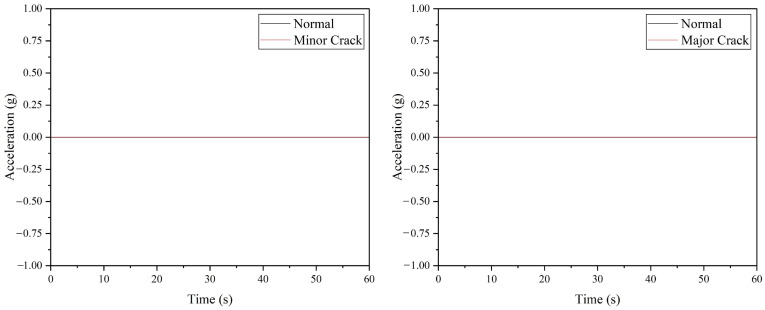
The acceleration values acquired from ADXL377 accelerometer for drone arm fault simulations.

**Figure 14:**
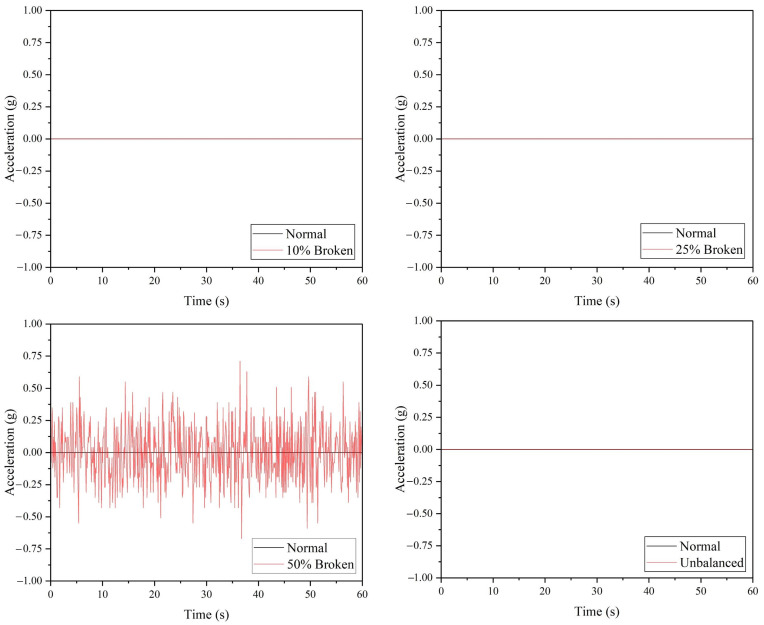
The acceleration values acquired from ADXL377 accelerometer for propeller fault simulations.

Referring to Figure 13, the acceleration values obtained from the ADXL377 accelerometer demonstrated the incapability of the MEMS sensor in detecting vibration induced by the minor and major crack in the drone arm. This is due to the sensor’s high measurement range (±200 g), which is more suitable for high vibration applications. For the propeller component, out of all the configurations, only 50% broken propeller can be detected by the ADXL377 accelerometer, where the maximum amplitude recorded is 0.71 g, as listed in Table 8. Figure 14 shows other faulty propeller cases generated 0 g vibration amplitude.

## 3. Proposed Anomaly Detection Prototype

**Figure 15:**
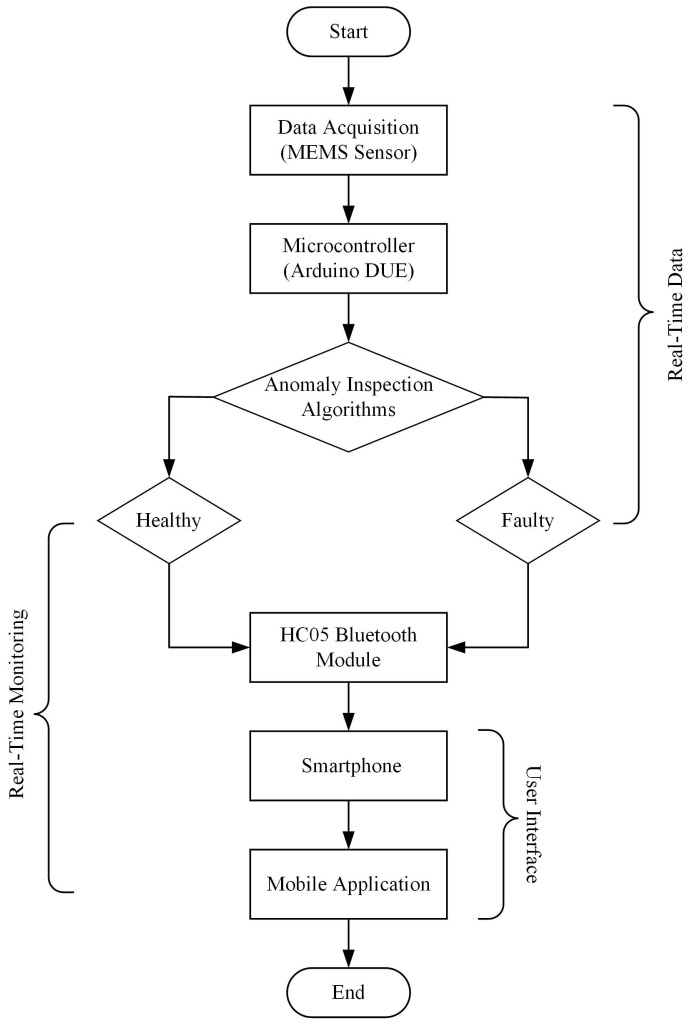
The block diagram of the proposed anomaly detection prototype.

The block diagram of the proposed framework is depicted in Figure 15. Firstly, vibration data are obtained from eight MEMS vibration sensors, where each sensor is installed at each drone arm. The advantages and disadvantages of the different MEMS vibration sensors in the real-time vibration-based anomaly inspection in drone are listed in Table 9. The MEMS sensor chosen for the final framework is the ADXL335 accelerometer due to its capability to detect various faulty cases and easy integration with the microcontroller. The microcontroller (Arduino DUE) records the data, and the anomaly inspection algorithms will compute the vibration data collected and provide the decision-making, whether the drone is in a healthy (no anomaly) or faulty state (anomaly present).

Referring to Table 6, a threshold value was set to differentiate the normal and faulty conditions based on the lowest maximum amplitude value of faulty conditions. Among the faulty conditions, a 10% broken propeller produced the lowest maximum amplitude value, which is 0.05 g. The drone is suspected of containing an anomaly if the acceleration data exceeds the threshold value. A drone must not exceed the threshold value to be considered healthy or normal. The drone’s state will be transmitted to the smartphone or tablet, and via the mobile application created, the user can inspect any anomaly in the drone in real-time.

**Figure 16:**
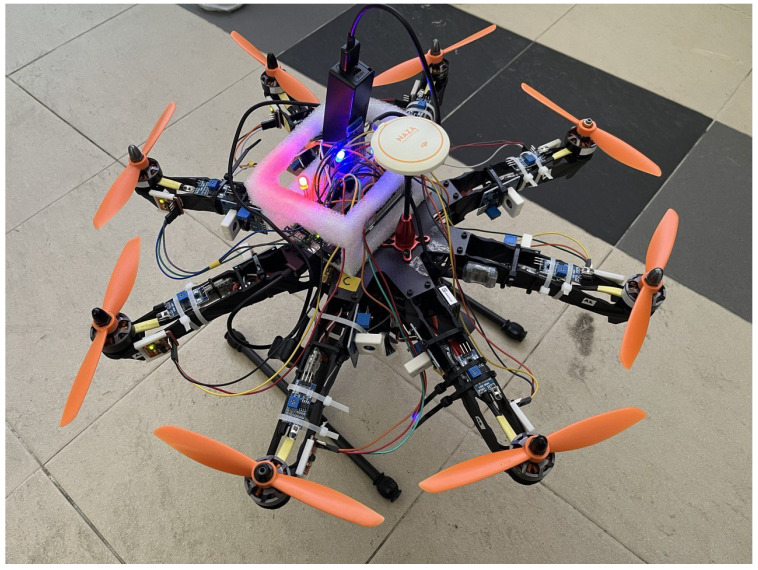
The proposed framework of early anomaly detection in drone.

**Figure 17:**
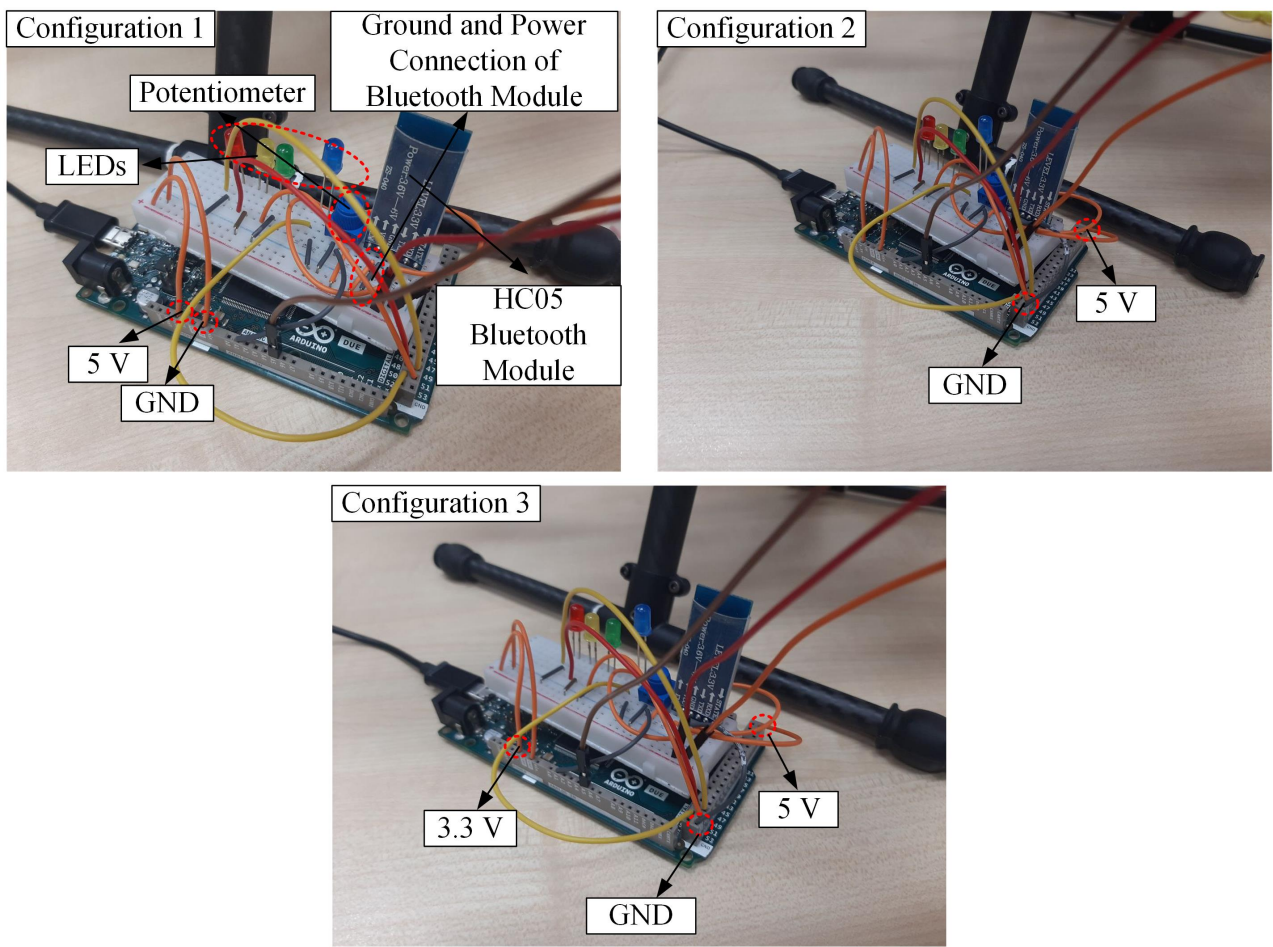
The drone arm configurations involved in the experiment.

Figure 16 portrays our proposed framework, and the role of each component involved in the framework is listed in Table 9. This framework is practical to be attached on top of the drone during flying because the overall weight is approximately 129 g, which is below the maximum payload of Storm Drone 8 (400 g). In terms of sensor data, it is expected that the MEMS sensor that shares an electrical connection with LEDs, potentiometer, and HC05 Bluetooth module will produce noisier data than a single sensor. Firstly, three different electrical connection configurations are tested to identify the different levels of noise obtained from one MEMS sensor, as shown in Figure 17. For Configuration 1, all the related components are connected to the same ground and 5 V power pin of Arduino DUE. In Configuration 2, the HC05 Bluetooth module is separated from other components by connecting it to other ground and 5 V power pins. Finally, we changed the power pin of other components besides the HC05 Bluetooth module from 5 V to a 3.3 V power pin.

**Figure 18:**
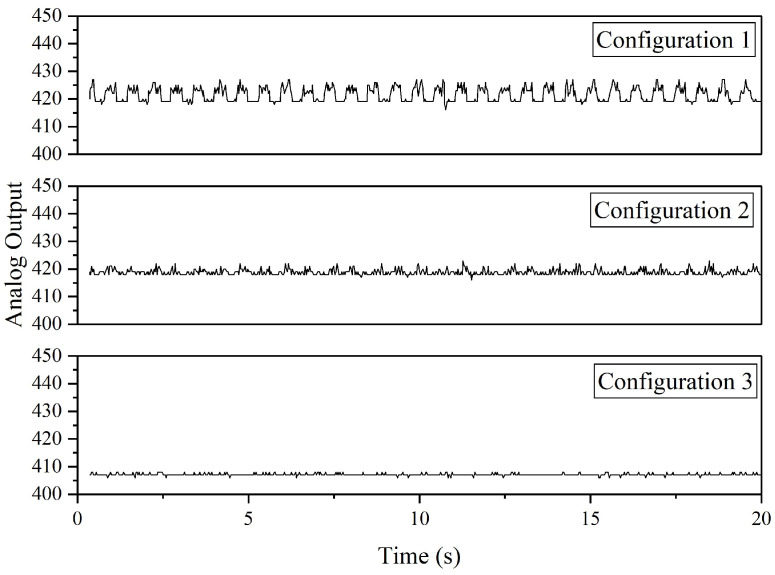
The analog outputs of ADXL335 accelerometer (one sensor connection).

Figure 18 shows the analog output of Configurations 1, 2, and 3. For the purpose of analyzing noise, these data are obtained when the drone is in “static mode”, where the motors are not spinning. Based on the results, Configuration 3 produced the lowest noise where the analog outputs recorded are constantly at 407 and sometimes fluctuated between 406 and 407. Configuration 1 contains the highest noise as the recorded analog output fluctuated between 419 and 427. In terms of standard deviation, similar results are obtained, in which Configuration 1 produced the highest value (2.5875), followed by Configuration 2 (1.0614) and 1 (0.398). In Configuration 1, integrating the HC05 Bluetooth module into the same ground and power connection with the ADXL335 accelerometer will disrupt the sensor circuit that is converting the motion into a voltage signal. The analog outputs are obtained from mapping the input voltage (5 V or 3.3 V) into integer values between 0 and 1023; therefore, due to the 3.3 V feature of the Arduino DUE, it is suspected that supplying 5 V to the power line that connects the sensor with LEDs and potentiometer may disrupt the mapping process.

**Figure 19:**
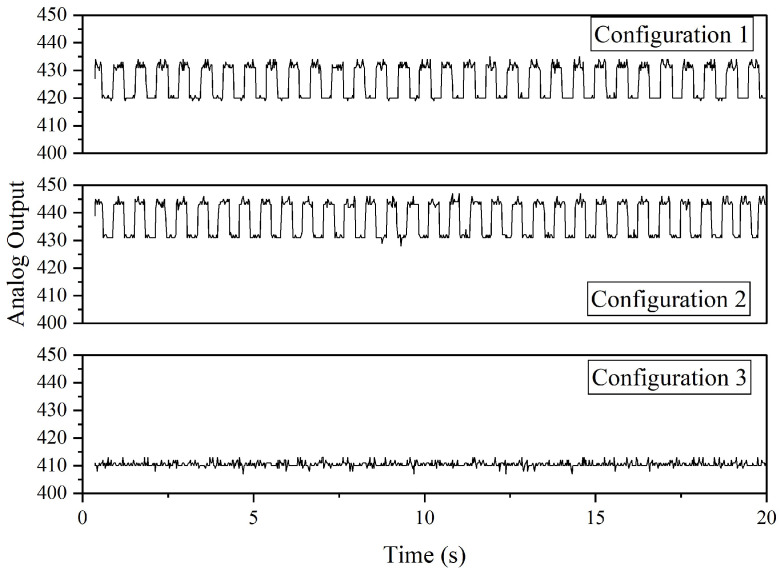
The analog outputs of one ADXL335 accelerometer when connected with other sensors.

**Figure 20:**
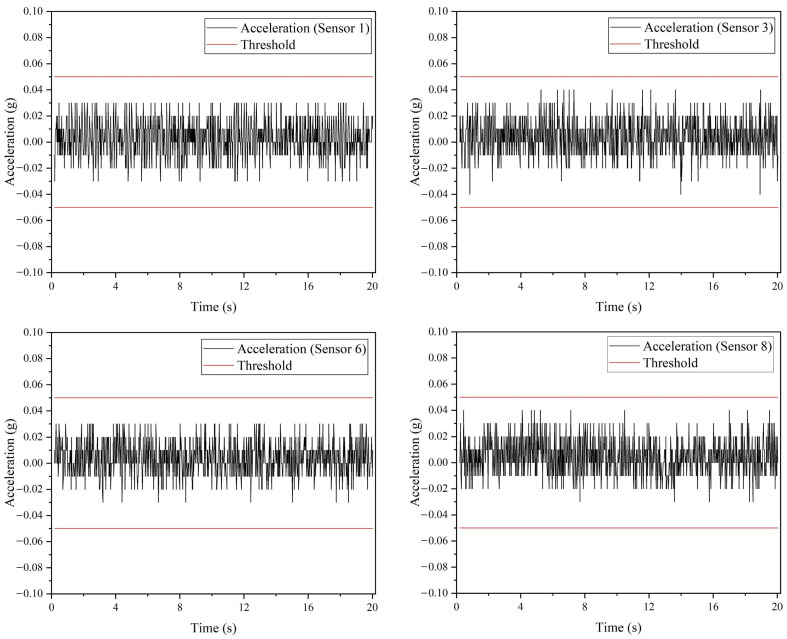
The normal vibration data recorded from four ADXL335 accelerometers installed on drone arms.

Next, the remaining seven ADXL335 accelerometers are integrated into the hardware to determine the effect of noise on the sensor data when all the sensors are connected. A similar case can be observed where Configuration 3 produced the lowest noise compared to Configuration 1 and 2, as depicted in Figure 19. The analog outputs are constantly at 410 and sometimes fluctuate between 408 and 412; however, in contrast to the one sensor case (Figure 18), there is a high fluctuation between 431 and 446 in the analog output recorded from Configuration 2. In terms of standard deviation, Configuration 1 produced the highest standard deviation value of 6.2276 compared to Configuration 2 (5.9192) and 3 (0.9493). Based on Configuration 3, the normal vibration levels recorded from four of eight ADXL335 accelerometers installed on the drone arm are depicted in Figure 20.

## 4. Mobile Application

**Figure 21:**
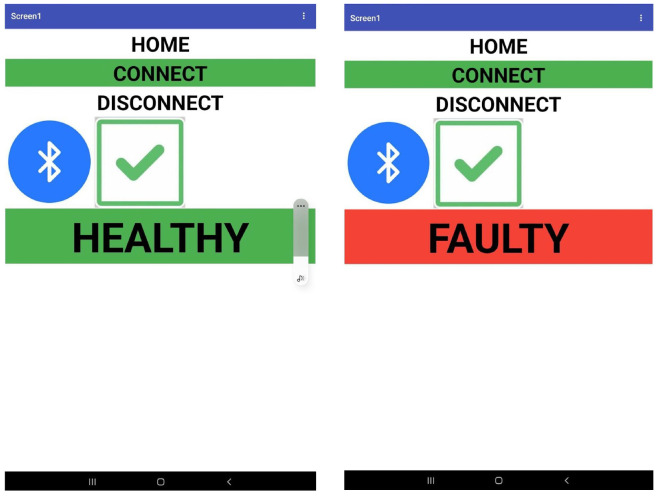
The user interface of the real-time anomaly inspection of drone mobile application.

Since users are understandably unfamiliar with the outputs in decimal form, a mobile application is specifically created for better and easier monitoring using Kodular, an open-source web application that allows users to design software applications for the Android operating system. By connecting the smartphone with the HC05 Bluetooth module, the anomaly inspection output is displayed on the smartphone, indicating a healthy or faulty drone condition, as shown in Figure 21.

## 5. Conclusions

This paper presents the real-time vibration-based early anomaly inspection in drones, focusing on the propeller and drone arm components. By investigating the performance of several MEMS vibration sensors, it was demonstrated that the ADXL335 and ADXL345 accelerometers could detect all the faulty cases based on the raw acceleration data. The SW420 vibration sensor is unable to detect a minor crack in the drone arm, 10% broken propeller, and 25% broken propeller, whereas the ADXL377 accelerometer can only detect 50% broken propeller. Based on the experimental results, 50% and 10% broken propeller generate the highest vibration, respectively. In the final framework, the ADXL335 accelerometer is chosen over the ADXL345 accelerometer because it is easier to integrate eight ADXL335 accelerometers with Arduino DUE compared to the ADXL345 accelerometer. The drone’s condition is classified into healthy and faulty states, which users can monitor through the mobile application. It is important to note that here all the signals were analyzed without post-processing or feature extraction methods. The performance of MEMS sensors can be improved by implementing signal processing techniques such as fast Fourier and wavelet transform. Currently, our framework is limited to fault detection, where it is unable to isolate the kinds of faults detected. This work can be further extended for fault classification application, by incorporating artificial intelligence or other fault recognition methods to further classify the fault into their types and severity.

## Figures and Tables

**Table 1:** The technical specification of the SW420 vibration sensor.

No.	Specification	Information
1	Dimension	3.2 cm × 1.4 cm
2	Weight	2 g
3	Voltage used	3.3 V to 5 V
4	Output	Analog and digital
5	Operating current	15 mA

**Table 2:** The vibration data recorded using SW420 vibration sensor.

Configuration	Maximum Amplitude	Average Amplitude
Normal (Drone arm and propeller)	0	0
Minor crack (Drone arm)	0	0
Major crack (Drone arm)	3495	2370
10% Broken (Propeller)	0	0
25% Broken (Propeller)	0	0
50% Broken (Propeller)	16,832	14,464
Unbalanced (Propeller)	368	244

**Table 3:** The technical specification of the ADXL345 accelerometer.

No.	Specification	Information
1	Dimension	2 cm × 1.5 cm
2	Weight	1 g
3	Voltage used	2 V to 3.6 V
4	Output	Digital
5	Operating current	23 μA
6	Sensitivity	256 LSB/g
7	Linear range	±2 g to ±16 g
8	Bandwidth	0.1 Hz to 3200 Hz

**Table 4:** The vibration data recorded using ADXL345 accelerometer.

Configuration	Maximum Amplitude (g)	Average Absolute Amplitude (g)
Normal (Drone arm and propeller)	0.71	0.18
Minor crack (Drone arm)	0.89	0.22
Major crack (Drone arm)	1.82	0.46
10% Broken (Propeller)	0.83	0.22
25% Broken (Propeller)	1.65	0.55
50% Broken (Propeller)	3.46	1.5
Unbalanced (Propeller)	−1.72	0.41

**Table 5:** The technical specification of the ADXL335 accelerometer.

No.	Specification	Information
1	Dimension	2.3 cm × 2 cm
2	Weight	1 g
3	Voltage used	2.5 V to 6 V
4	Output	Analog
5	Operating current	300 μA
6	Sensitivity	300 mV/g
7	Linear range	±3 g
8	Bandwidth	0.5 Hz to 1600 Hz (X and Y axes), 0.5 Hz to 550 Hz (Z axis)

**Table 6:** The vibration data recorded using ADXL335 accelerometer.

Configuration	Maximum Amplitude (g)	Average Absolute Amplitude (g)
Normal (Drone arm and propeller)	0.03	0.0068
Minor crack (Drone arm)	0.07	0.016
Major crack (Drone arm)	0.09	0.025
10% Broken (Propeller)	0.05	0.008
25% Broken (Propeller)	0.08	0.03
50% Broken (Propeller)	0.15	0.068
Unbalanced (Propeller)	0.06	0.018

**Table 7:** The vibration data recorded using ADXL377 accelerometer.

Configuration	Maximum Amplitude (g)	Average Absolute Amplitude (g)
Minor crack (Drone arm)	0	0
Major crack (Drone arm)	0	0
10% Broken (Propeller)	0	0
25% Broken (Propeller)	0	0
50% Broken (Propeller)	0.71	0.18
Unbalanced (Propeller)	0	0

**Table 8:** The technical specification of the ADXL377 accelerometer.

No.	Specification	Information
1	Dimension	2 cm × 2 cm
2	Weight	1 g
3	Voltage used	1.8 V to 3.6 V
4	Output	Analog
5	Operating current	300 μA
6	Sensitivity	6.5 mV/g
7	Linear range	±200 g
8	Bandwidth	0.5 Hz to 1300 Hz (X and Y axes), 0.5 Hz to 1000 Hz (z-axis)

**Table 9:** The components involves in the proposed drone anomaly inspection system.

Component	Role	Weight
Arduino DUE	Compute the anomaly inspection algorithm based on the data from MEMS sensors	36 g
HC05 Bluetooth module	Transmit the output from the anomaly inspection algorithm to the mobile application in the smartphone	2 g
Eight ADXL335 accelerometers	Supply vibration data to Arduino DUE	8 g
Potentiometer	Turn on and off the proposed framework	0.5 g
Blue LED	Will light up to indicate the ON mode of the system	0.2 g
Green LED	Will light up to indicate the healthy drone condition	0.2 g
Yellow LED	Will light up to indicate the error in the system	0.2 g
Red LED	Will light up to indicate the faulty drone condition	0.2 g
Power bank (3000 mah)	Supply power to the overall system	54 g
Mini breadboard	Provide electrical connections between electronic components	18 g
Framework cover	Provide cover to the framework	10 g

## Data Availability

The data presented in this study are available on request from the corresponding author.

## Abbreviations

The following abbreviations are used in this manuscript:

MEMS	Microelectromechanical Systems
UAV	Unmanned Aerial Vehicle
ESC	Electronic Speed Controller
NN	Neural Network
DWT	Discrete Wavelet Transform
SVM	Support Vector Machine
IMU	Inertial Measurement Unit
CNN	Convolutional Neural Network
ATV	Autonomous Transfer Vehicle
LCD	Liquid-crystal Display
LED	Light Emitting Diode
IC	Integrated Circuit
I2C	Inter-Integrated Circuit
SPI	Serial Peripheral Interface

## Appendix A

**Table A1:** The advantages and disadvantages of different MEMS vibration sensors in the real-time vibration-based anomaly inspection in drone application.

MEMS Vibration Sensors	Advantages	Disadvantages
SW420	More sensors can be integrated into the microcontroller compared to accelerometers Cheap	Not suitable for early drone anomaly inspection application Output has no physical unit
ADXL345	Suitable for early drone anomaly inspection application Low power consumption	Requires I2C communication protocol, which limits the number of sensors that can be applied Slightly more complicated to use compared to ADXL335 and ADXL377
ADXL335	Suitable for early drone anomaly inspection application Easy to use	Expensive Narrow g range
ADXL377	Wide g range Easy to use	Not suitable for early drone anomaly inspection application Expensive
